# Application of Fourier-Galois Spectra Analysers for Rotating Image Analysis

**DOI:** 10.3390/polym17131791

**Published:** 2025-06-27

**Authors:** Dina Shaltykova, Kaisarali Kadyrzhan, Ibragim Suleimenov, Gaini Seitenova, Eldar Kopishev

**Affiliations:** 1 National Engineering Academy of the Republic of Kazakhstan, Almaty 050010, Kazakhstan; shaltykova.d@mail.ru (D.S.); esenych@yandex.kz (I.S.); 2 Department of Telecommunication Engineering, Institute of Communications and Space Engineering, Gumarbek Daukeev Almaty University of Power Engineering and Communications, Almaty 050040, Kazakhstan; kaisarali1997ss@gmail.com; 3 Department of Chemistry, Faculty of Natural Sciences, L.N. Gumilyov Eurasian National University, Astana 010000, Kazakhstan

**Keywords:** circular images, algebraic extensions, rheological properties of liquids, Galois fields, Fourier–Galois transformation, Stokes method, digital logarithm

## Abstract

It is shown that the analysis of rotating circular images containing *n* = 2^p−1^ pixels, the state of which is described by variables of binary logic, and *p* is an integer, is expedient to carry out using digital spectra obtained using the Fourier–Galois transformation, and the basis corresponding to such a transformation is formed by the method of classical algebraic extensions of the main Galois field *GF*(2) corresponding to binary logic. It is shown that the use of Fourier–Galois spectra makes it possible to reduce the analysis of a rotating image to the analysis of a still image by using the operation of digital logarithm. It is shown that the proposed approach is of interest, including from the point of view of improving equipment designed to study the rheological properties of liquids, in particular, polymer solutions in which non-trivial branched structures are formed. In this case, the use of the proposed approach provides an opportunity to modernize the classical method of measuring Stokes viscosity, focused on the study of mechanochemical reactions. The design of a viscometer that implements the proposed approach has been developed. In it, a digital image is formed by a set of optoelectronic pairs that track the circular motion of the ball in a cuvette driven by rotation. The electronic circuits of this type of viscometer are based on a Fourier–Galois spectrum analyser and a digital logarithm operation. The possibilities of generalizing the proposed approach to the analysis of rotating images of other types are considered.

## 1. Introduction

Works devoted to the creation of pattern recognition methods are widely represented in the current literature [1,2,3]. Such methods are used in the analysis of various types of human activity [4,5], in health care [6,7], to improve sensory and measuring equipment [8,9], etc. Pattern recognition is increasingly used in the field of chemistry as well [10,11].

Image recognition methods can be used, among other things, to study systems in which relatively simple images are formed, for example, rotating images [12,13]. One example here is the development of laboratory equipment designed to measure viscosity [14]. In the cited work, it was proposed to study the viscosity of solutions by recording the images that occur during the circular motion of the ball in the liquid under study.

It should be noted that measurements of the viscosity of polymer solutions have traditionally been one of the main methods of their experimental study [15,16,17]. One of the most common methods for measuring viscosity is the Stokes method, based on determining the velocity of a ball in a viscous liquid. Such equipment has been used in particular in [18,19].

There are numerous attempts to modernize the tools of rheological research [20], but they are usually associated with the technical improvement of existing methods of viscosity measurement, which include capillary viscometers of various modifications [21,22], rotary viscometers [23,24,25], etc.

Various modifications of the Stokes method are known, for example, [26,27], where additional parameters such as ball surface properties or the effects of boundary layer instability were taken into account to enhance the accuracy and expand the applicability of the method to complex fluids. In the paper [28,29], a modification of this method was also proposed, suitable not only for measuring the viscosity of a liquid, but also for studying mechanochemical processes that occur in a liquid due to the movement of a ball immersed in it at a sufficiently high speed. On this basis, a patent was also obtained. The patent is provided in the Supplementary Materials.

Such an approach, in particular, is of interest for the study of a relatively new class of reaction products between water-soluble polymers—hydrophilic and hydrophobic–hydrophilic associates [30]. Their study is now of particular interest. Namely, in the paper [31], based on the results of [32,33], the hypothesis was substantiated, according to which the universe can be considered as an analogue of a neural network. This hypothesis clearly correlates with the conclusions made in [30,34], where it was shown that any solution of a partially dissociating polymer is also a direct analogue of a neural network, more precisely, a Hopfield neural network.

More precisely, the methodological connection between the above-cited works was demonstrated in [35], where it was shown that it is the neural network properties of macromolecule solutions that make it possible to substantiate the existence of a mechanism of prebiological evolution, free from the shortcomings inherent in theories going back to the Darwinian point of view.

It should also be noted that the conclusions of the works [31,35] also clearly correlate with the results of the works [36,37], in which it was shown that society as a whole is also a direct analogue of a neural network. Consequently, there is every reason to believe that the consideration of complex systems of a very different nature from the point of view of their analogy with neural networks can become one of the most important tools for interdisciplinary cooperation, and possibly for general methods for studying complex systems regardless of their nature. Methodological prerequisites for such a conclusion also exist. In particular, it was shown in the works [34,38,39,40] that the behaviour of complex systems is determined not so much by the nature of their elements as by the architecture of connections between them.

However, the conclusion about the neural network nature of polymer solutions was made in [30,34] on the basis of a limited range of experimental data, mainly obtained by turbidimetry methods.

Given the high methodological significance of this conclusion, it seems very important to develop additional methods for studying polymer solutions, which returns to the question of modernizing existing viscometry tools. Moreover, viscometry should be considered as one of the most promising tools for studying the neural network properties of polymer solutions.

Indeed, in the cited works, as well as in [41], it was shown that the product of the interaction of an ionic and non-ionic polymer in a solution is, among other things, a hydrophilic interpolymer associate (GIA). It is an intermediate product between classical interpolymer complexes, polymer gels, and true solutions. The GIA is a network that exists in a dynamic mode, i.e., the hydrogen bonds that stabilize it arise and then break again. Therefore, the motion of the ball that is used in the Stokes method to measure viscosity is, generally speaking, a mechanochemical process (in the medium where GIAs or similar reaction products are formed).

This makes it necessary to modernize the Stokes method, focusing on the use of a ball moving at different speeds. A certain step in this direction was made, among other things, in [42,43]. However, the main task that was solved in the cited work was to ensure a reduction in operating costs when using viscometers, associated mainly with the need for thorough flushing of measuring units. For this in particular, it was proposed to switch to the use of disposable measuring cells.

In this work, an approach is proposed that creates the basis for further modernization of the Stokes method, aimed not only and not so much at studying the viscosity of polymer solutions, but at studying their mechanochemical properties in the above context. At the same time, the approach proposed, in particular, in [42], is also employed, which implies that the cost of the developed devices can be significantly reduced through the most extensive use of modern software solutions.

It should also be emphasized that this method is also suitable for solving other similar problems in which it is necessary to reduce a rotating image to a still one.

## 2. Materials and Methods

The proposed approach is based on the application of Fourier–Galois transformations to the analysis of a rotating ring-shaped image represented as a sequence of logical values (0 and 1), corresponding to a discrete set of points into which the ring is divided. This set is associated with a function *f(m)* defined over the Galois field GF(2). Such a function can be expanded into a finite series using sequences that form an orthogonal basis in the Galois field GF(2*^k^*), where *k* is an integer, under the condition that *q* = 2*^k^* − 1, with *q* being the number of elements into which the ring image is divided. In this case, the sequences forming the orthogonal basis are expressed by the following formulas:

(1) $$g_{n}=\left(u_{n1},u_{n2},\ldots ,u_{nN}\right)$$

where is

(2) $$u_{nm}={\left(\theta^{n}\right)}^{m}=\theta^{nm}$$

θ is a primitive element of the Galois field GF(2^k^), i.e., an element whose powers from 1 to 2^k^ − 1 exhaust the set of all non-zero elements of the field under consideration.

The Fourier–Galois series decomposition has the form

(3) $$f(m)=\sum_{n}a_{n}g_{n}\left(m\right)$$

where the coefficients $a_{n}$ are calculated using the formula

(4) $$a_{n}=q^{-1}\sum_{n}f\left(m\right)g_{n}^{*}(m)$$

where the sequence $g_{n}^{*}(m)$ is the sequence conjugate to $g_{n}(m)$, and $q^{-1}\;$denotes the multiplicative inverse of *q* in the sense of arithmetic over the Galois field GF(p^k^).

Sequence elements $u_{nm}^{*}$ satisfy the condition

(5) $$u_{nm}^{*}u_{nm}=1$$

Condition (5) is met if the elements of the sequences $u_{nm}^{*}$ are expressed by the formula:

(6) $$u_{nm}^{*}={\left(\theta^{q-n}\right)}^{m}=\theta^{-nm}$$

where $q=2^{k}-1$.

In this case,

(7) $$u_{nm}^{*}u_{nm}={\left(\theta^{m}\right)}^{q-n}{\left(\theta^{m}\right)}^{n}={\left(\theta^{m}\right)}^{q}=1$$

Relation (7) holds because an arbitrary non-zero field element $GF(2^{k})$ satisfies the equation

(8) $$x^{q}-1=0$$

The main feature of expansion (3), when applied to functions that take discrete values at discrete points, is as follows. The number of elements N in the sequences $g_{n}$ must either coincide with the number of non-zero elements q in the employed field, or q must be a multiple of N, i.e., N must divide q exactly. This imposes specific constraints on the functions that can be used, although such constraints are not always critical.

As will become clear later, the use of Fourier–Galois transformations effectively enables the conversion of a rotating image into a stationary one due to the relationship between the spectra of such images.

## 3. Results

### 3.1. A General Approach to the Analysis of Rotating Images Using Spectra in Binary Galois Fields

The principle of reducing rotating images to still images is based on the following property of the Fourier–Galois transformation. Let us consider the following expression, which, among other things, corresponds to the study of a rotating image.

(9) $$a_{n}=q^{-1}\sum_{n}f\left(m-m_{0}(t)\right)u_{n}^{*}(m)$$

This formula reflects the following fact: the analysed image rotates, yet it remains unchanged. Therefore, it is described by the same function $f\left(m\right)$ as the stationary image, with the only difference being the appearance of a phase-shift analogue that depends on time. Let us now take into account the key property of the function $u_{n}^{*}(m)$, which follows from Equation (6).

(10) $$u_{nm}^{*}=\theta^{-nm}=\theta^{-n\left(m-m_{0}(t)\right)}\theta^{nm_{0}(t)}$$

Then Formula (9) can be written in the following form.

(11) $$a_{n}=q^{-1}\theta^{nm_{0}(t)}\sum_{n}f\left(m-m_{0}(t)\right)\theta^{-n\left(m-m_{0}(t)\right)}$$

Moving on to the summation index $m_{1}=m-m_{0}(t)$, we get

(12) $$a_{n}(t)=\theta^{nm_{0}(t)}a_{n}^{0}$$

This means, in particular, that in order to determine the rotation speed of an image, it is permissible to record changes in the phase multiplier over time. In addition, by excluding time-dependent phase multipliers from the spectrum, you can reveal a “frozen” picture. The exclusion of phase multipliers, which depend on time, can, in turn, be carried out using a digital logarithm operation.

Indeed, any non-zero element of the Galois field under consideration is representable in the following form.

(13) $$x=\theta^{s}$$

The representation (2) used above is a special case of this formula. The operation referred to as digital logarithm can be understood as the mapping of a field element x to the exponent s in the given formula. Applying this operation to Formula (12) yields an evident result.

(14) $$LD\left[a_{n}(t)\right]=nm_{0}\left(t\right)+LD\left[a_{n}^{0}\right]$$

where through LD is the digital logarithm operation.

It can be seen that the first term on the right side of Formula (14) is the same for all spectral components (with an accuracy of up to a factor of n). The second term, if the image is indeed rotating, does not depend on time.

Consequently, it is possible to set the task of excluding the term $nm_{0}\left(t\right)$ by using a computational algorithm, which, among other things, is implemented by circuitry. A specific example of solving such a problem is in this work. This example, among other things, shows that it is convenient to solve the problem of analysing rotating images of any type (provided that they are reduced to a discrete form) through the use of Fourier–Galois transformations.

### 3.2. The Design of the Proposed Type Viscometer and the Principle of Its Operation

It should be emphasized once again that the proposed design, on the one hand, solves a particular problem (the study of the viscosity of polymer solutions taking into account possible mechanochemical processes). On the other hand, the proposed approach allows us to show that the operation of digital logarithm can become a very effective means of studying rotating images (including in terms of identifying the image as rotating).

**Figure 1:**
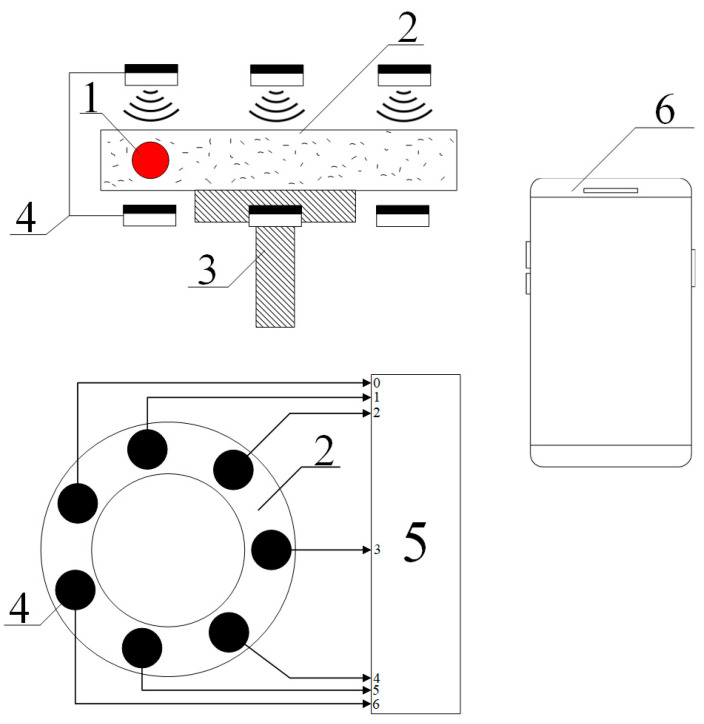
Functional diagram of a viscometer based on the recognition of a discrete ring image.

The functional diagram of the proposed device designed to measure viscosity and identify the occurrence of concomitant mechanochemical effects is presented in Figure 1.

The principle of operation of the proposed type of viscometer, as in the case considered, in particular, in [28], is based on the induced rotation of the ball (1) located inside the measuring cell (2). This cell is filled with the test fluid and is located on a rotating bed (3). The ball is involved in a circular motion due to high enough viscosity of the solution. Seven optoelectronic pairs (4) are used to record the rotation pattern of the ball, allowing the capture of a circular image split into seven pixels. Optoelectronic pairs (4) are set to threshold actuation, i.e., at the output of each of them a signal is generated that corresponds to either a logical zero or a logic one. Signals taken from pairs (4) are fed to the primary signal processing unit (5), which performs, among other things, the operation of digital logarithm. All other operations that are computational procedures, as shown in [42,43], should be performed using a program installed on the user’s smartphone or iPhone (6). Data transmission from the unit (5) to the smartphone is carried out using a Bluetooth module on phone (6).

We emphasize that in this case it really makes sense to talk about the study of the image, especially if we take into account the conclusions made in the works [35,36]. Namely, a priori mechanochemical effects can be considered quite weak, so it is advisable to use conditions when the system under study is on the verge of a phase transition to detect them. Such conditions are identified from the cited works. In the same place, as well as in many other works, in particular, [44,45,46], it is shown that the phase transition is accompanied by a change in the turbidity (optical density) of the solution. Therefore, by adjusting the lighting, it is possible to ensure that the “trace” of the ball moving along the circumference of the cuvette will be visible, due to the fact that its impact on the solution has led to a change in optical density due to the course of mechanochemical reactions.

It should also be emphasized that the choice of the number of optoelectronic pairs was made, first, based on the criterion formulated in the Section 2, i.e., the number 7 corresponds to the formula $q=2^{k}-1\;$for k=3; second, it aligns with the arguments presented in [47], according to which the selection of a specific Galois field should be guided by the specifics of the particular problem.

### 3.3. The Set of Basic Functions Used

In this work, a field $GF(2^{3})$ is used, the number of non-zero elements of which is 7, which corresponds to the design of a viscometer considered above, which is also intended for the study of mechanochemical processes in solutions in which hydrophobic–hydrophilic or hydrophilic associates are formed.

A specific type of field $GF(2^{3})$ element is obtained using the following irreducible equation given over the field GF(2):

(15) $$x^{3}+x+1=0$$

A direct verification shows that Equation (15) has no solutions in the field GF(2), which contains only two elements: 0 and 1. At the same time, Equation (15) is of third order, which allows for the construction of an algebraic extension of the field GF(2) of the following form:

(16) $$z=z_{2}\theta^{2}+z_{1}\theta +z_{0}$$

where θ is a formal root of the irreducible Equation (15), which can also be interpreted as a logical imaginary unit [48], and $z_{i}$ are coefficients from the field GF(2).

This construction fully corresponds to the classical method of building algebraic extensions of the base Galois field, which are widely used in modern information technologies [49,50]. The element θ is primitive, which can be demonstrated directly, while simultaneously obtaining the explicit form of the remaining elements of the field GF(2).

Since the extension is of degree 3, the elements θ and $\theta^{2}\;$are linearly independent. The third power of θ can be expressed in terms of lower-order powers in accordance with Equation (15) as:

(17) $$\theta^{3}=\theta +1$$

By raising the element θ to integer powers, we get all the non-zero elements of the field under consideration (Table 1).

As follows from Formula (2), any element of the sequences $g_{n}$ coincides with one of the elements listed in Table 1. More precisely, these sequences are composed of powers of θ, arranged in an order determined by Formula (2) and relation (8), i.e., the product *nm* appearing in Formula (2) is computed modulo 7. The corresponding exponent values are given in Table 2.

Table 2 also takes into account the following nuance. By virtue of the relation (8), there is a

(18) $$u_{n7}={\left(\theta^{n}\right)}^{7}=\theta^{7n}=1$$

This means that one of the sequences forming the orthogonal basis consists entirely of elements equal to 1.

Based on Table 2, Table 3 has been constructed to display the basis sequences $g_{n}(m)\;$in binary representation. This representation corresponds to Formula (16), i.e., the rows of Table 3 show the values of the coefficients $z_{i}$ for each element of the basis sequence. Each segment of the table corresponds to a sequence with a specific number *n* (indicated in Table 3).

Each row in this table can be regarded as a discrete function $z_{i}^{n}(m)$. Analysis of the results presented in Table 3 shows that the basis sequences $g_{n}(m)$ generate exactly eight functions corresponding to $z_{i}^{n}(m)$. Formally, there are 18 such functions, but some of them are identical, as indicated in Table 3 by colour highlighting. These eight functions can be numbered, and the assigned numbers s are shown in the last column of Table 3.

Functions $z_{i}^{n}(m)$ are generated by sequences that form an orthogonal basis. Therefore, it makes sense to check these functions themselves for the fulfilment of orthogonality relations in the sense of calculations in the main Galois field. The results of such a check are presented in Table 4. 

The resulting table shows that if rows and columns numbered 4 and 8 are excluded, the remaining part corresponds to six out of the eight functions $z_{i}^{n}(m)$ that exhibit mutual orthogonality. This implies that the functions with indices 1, 2, 3, 5, 6, and 7 (as indicated in the last column of Table 3) form a complete basis over a segment containing seven cycles, provided that this set is supplemented by the function corresponding to the all-ones sequence. In this case, the seven cycles are represented by exactly seven basis functions that are mutually orthogonal.

**Figure 2:**
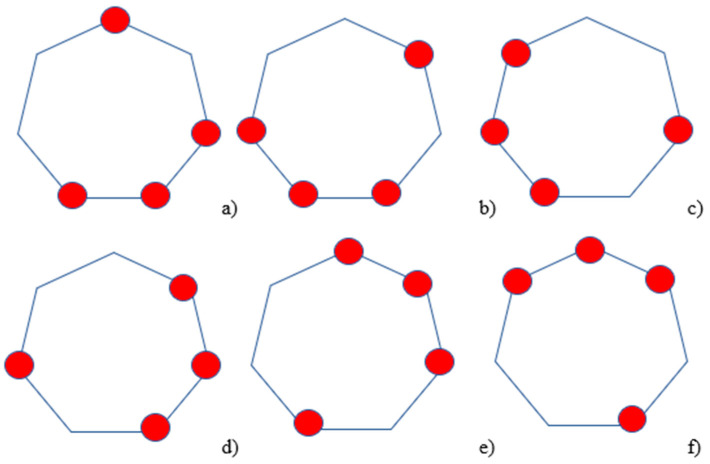
Illustration of the orthogonal basis in the field GF(2), derived from the orthogonal basis in the field GF(2^3^); The sequence of subfigures illustrates transformations of the orthogonal basis under various rotational operations: (**a**) the initial position (reference state), (**b**) a rotation by $\frac{2\pi }{7}\;$ clockwise, (**c**) a rotation by $\frac{4\pi }{7}\;$clockwise, (**d**) a rotation by $\frac{4\pi }{7}\;$ counterclockwise, (**e**) a rotation by $\frac{6\pi }{7}\;$ counterclockwise, and (**f**) a rotation by $\frac{6\pi }{7}\;$ clockwise.

The specific characteristics of the functions taking values in the base field GF(2), corresponding to the above-mentioned indices, are illustrated in Figure 2. The vertices of the heptagons marked with red circles correspond to values of 1; otherwise, the value is 0.

Hereafter, we will denote the functions corresponding to Figure 2 as $G_{i}$, where i=1,2,3,5,6,7. In addition to these, there also exist functions $G_{4}$ and $G_{8}$; however, according to the results presented in Table 4, they are linearly dependent on the others.

(19) $$G_{4}=G_{1}+G_{2},\;G_{8}=G_{5}+G_{6}$$

In these notations, the basic functions that take values in the field can be represented as $GF(2^{3})$

(20) $$g_{1}=G_{3}+\theta G_{1}+\theta^{2}G_{2}$$

(21) $$g_{2}=G_{3}+\theta G_{2}+\theta^{2}G_{4}=G_{3}+\left(\theta +\theta^{2}\right)G_{2}+\theta^{2}G_{1}$$

(22) $$g_{3}=G_{7}+\theta G_{8}+\theta^{2}G_{5}=G_{7}+\theta G_{6}+\left(\theta +\theta^{2}\right)G_{5}$$

(23) $$g_{4}=G_{3}+\theta G_{4}+\theta^{2}G_{1}=G_{3}+\theta G_{2}+\left(\theta +\theta^{2}\right)G_{1}$$

(24) $$g_{5}=G_{7}+\theta G_{6}+\theta^{2}G_{8}=G_{7}+\left(\theta +\theta^{2}\right)G_{6}+\theta^{2}G_{5}$$

(25) $$g_{6}=G_{7}+\theta G_{5}+\theta^{2}G_{6}$$

There is one more basis function, $g_{7}$, all of whose elements are equal to 1. The Fourier–Galois spectrum of the values $U_{n}$, recorded from the viscometer sensors, is thus given by the following formula:

(26) $$F_{m}=\sum_{n}U_{n}g_{m}\left(n\right)=\sum_{k}\alpha_{km}A_{k}$$

where the summation is carried out by the values of k=1,2,3,5,6,7.

The coefficients $\alpha_{km}$ essentially represent the elements of a matrix that relates the basis $g_{m}$ to the basis $G_{m}$, while $A_{k}$ represent the convolution of the basis functions $G_{m}$ with the sequence of binary variables describing the image under consideration (in the case of the viscometer example—the sequence of binary variables read from the outputs of the optoelectronic pairs). The explicit expressions for these quantities are as follows:

(27) $$A_{1}{=}_{\left(2\right)}U_{1}+U_{3}+U_{4}+U_{5}$$

(28) $$A_{2}{=}_{\left(2\right)}U_{2}+U_{4}+U_{5}+U_{6}$$

(29) $$A_{3}{=}_{\left(2\right)}U_{1}+U_{5}+U_{6}+U_{7}$$

(30) $$A_{5}{=}_{\left(2\right)}U_{2}+U_{3}+U_{4}+U_{6}$$

(31) $$A_{6}{=}_{\left(2\right)}U_{1}+U_{2}+U_{3}+U_{5}$$

(32) $$A_{7}{=}_{\left(2\right)}U_{1}+U_{2}+U_{4}+U_{7}$$

The values of the coefficients $\alpha_{km}$ are presented in Table 5.

Table 5, among other things, emphasizes that the values of each spectral component can be presented in a form similar to (16)

(33) $$F_{m}=a_{2m}\theta^{2}+a_{1m}\theta +a_{0m}$$

where $a_{im}$ are expressed in terms of $\alpha_{km}$ and $A_{k}$ in accordance with Formula (26).

Rather

(34) $$a_{im}=\sum_{k}\beta_{ikm}A_{k}$$

where the summation is also carried out over the values k=1,2,3,5,6,7, and the values of the coefficients $\beta_{ikm}$, where i=0,1,2, are presented in Table 6.

**Figure 3:**
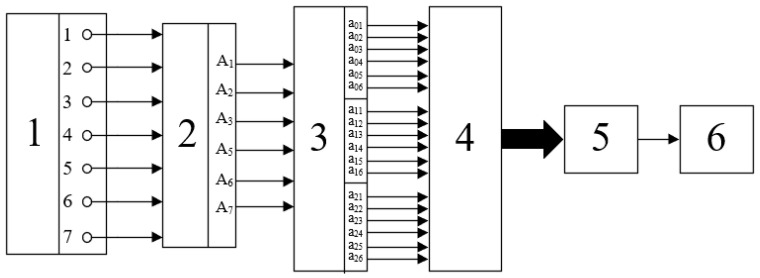
Rotary image analyser block diagram.

The values $a_{im}$ are binary variables and can be obtained by electronic means. The functional diagram of the Fourier–Galois spectrum analyser built on this basis is shown in Figure 3. The circuit also includes a digital logarithm unit, as discussed below. This scheme also serves as an electronic unit that ensures the operation of the viscometer of the proposed type.

The system includes a set of components that generate binary signals corresponding to the image registration using optoelectronic pairs (1); a block (2) for primary spectrum computation (i.e., a unit that computes the binary values $A_{k}$); a block (3) that calculates the values $a_{im}$ for all indices m; a switch (4); a digital logarithm block (5); and a Bluetooth module (6).

**Figure 4:**
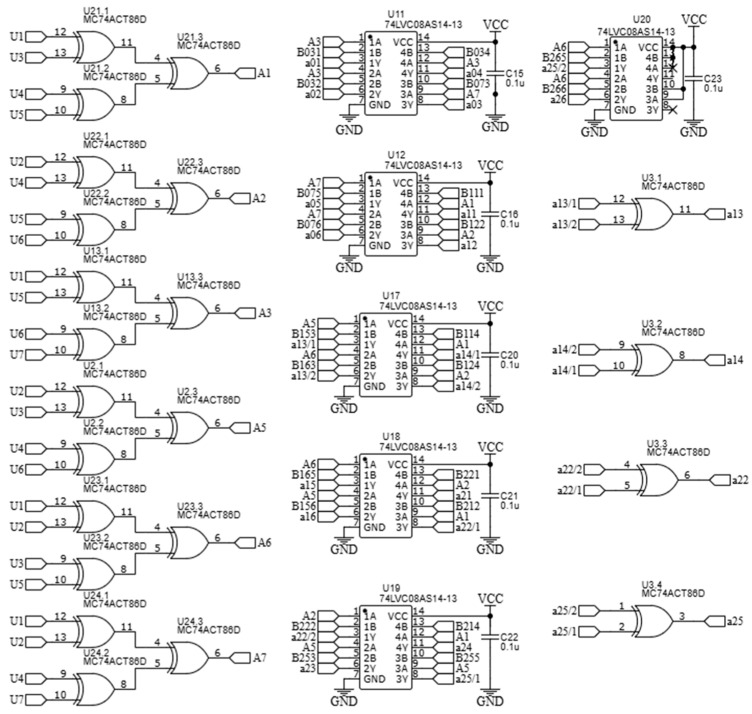
Schematic diagram of the Fourier–Galois spectrum analyser.

Block (2) has six inputs and six outputs, while block (3) has six inputs and eighteen outputs. The design of these blocks is relatively simple, as they are entirely based on logic elements that perform modulo-2 addition. Their overall circuit diagram is shown in Figure 4; this circuit essentially implements the Fourier–Galois spectrum computation in the field under consideration.

The inputs of elements U21, U22, U13, U2, U23, and U24—which are logic gates implementing the “exclusive OR” operation—are connected to the outputs of the optoelectronic pairs, corresponding to the binary values U_1_–U_7_. At the outputs of these elements, binary values A_k_ are generated, in accordance with Formulas (27)–(32). Subsequently, the obtained binary values A_k_ are fed into the inputs of elements U11, U12, U17, U18, U19, and U20, which perform the logical AND operation, as well as element U3, which also performs the “exclusive OR” operation. These components together carry out the computation of the elements $a_{im}$.

The switch sequentially transmits signals corresponding to the three values of $a_{im}$ for each spectral component to the digital logarithm block. The use of a switch is justified, as the operating speed of the employed logic elements significantly exceeds the typical rotation speeds of the images—at least in the case of the viscometer under consideration.

The rationale for applying the digital logarithm operation—which corresponds to mapping to the powers of the primitive element representing the elements of the given Galois field—is based on the following considerations. First, representing spectral components in terms of elements obtained via algebraic field extension (i.e., involving θ and its square) is not always convenient. Second, as follows from Formula (14), it is more appropriate to analyse a rotating image by converting to the powers of the primitive element. In the case under consideration, the digital logarithm operation can be defined explicitly, as shown in Table 7.

Let us obtain in an explicit form algebraic formulas that allow us to perform a digital logarithm operation corresponding to Table 7. For this purpose, it is advisable to use the Zhegalkin polynomial, which for the special case under consideration is convenient to represent in the following form.

(35) $$b_{i}=\sum_{k}\alpha_{ik}\sigma_{k}\left(a_{1},a_{2},a_{3}\right)$$

Here, $\sigma_{k}\left(a_{1},a_{2},a_{3}\right)$ is a function that is non-zero only for the specific combination of binary variables $a_{i}$ corresponding to the integer k; the coefficients $\alpha_{ik}$ correspond to the operation represented in Table 6. Formula (35) allows the value of the binary variable $b_{i}$ to be expressed as an algebraic function of all three values $a_{i}$.

In particular, for the coefficient $b_{2}$, this formula takes the following form (in this and subsequent formulas, calculations are performed modulo 2):

(36) $$b_{2}=\sigma_{4}+\sigma_{5}+\sigma_{6}=\left(a_{0}+1\right)a_{1}a_{2}+a_{0}a_{1}a_{2}+a_{0}\left(a_{1}+1\right)a_{2}$$

This formula contains three terms: $\sigma_{4}$, $\sigma_{5}$, $\sigma_{6}$. As can be seen from Table 7, $b_{2}$ is non-zero only for these specific values of k.

Through simple transformations, Formula (36) can be rewritten in a form that is convenient for implementation as an electronic circuit.

(37) $$b_{2}=a_{2}\left(1+\left(a_{0}+1\right)\left(a_{1}+1\right)\right)$$

Similarly

(38) $$b_{1}=\left(a_{0}+1\right)\left(a_{1}+1\right)a_{2}+a_{0}a_{1}\left(a_{2}+1\right)+a_{0}\left(a_{1}+1\right)a_{2}$$

(39) $$b_{0}=\left(a_{0}+1\right)a_{1}\left(a_{2}+1\right)+a_{0}a_{1}\left(a_{2}+1\right)+a_{0}a_{1}a_{2}$$

**Figure 5:**
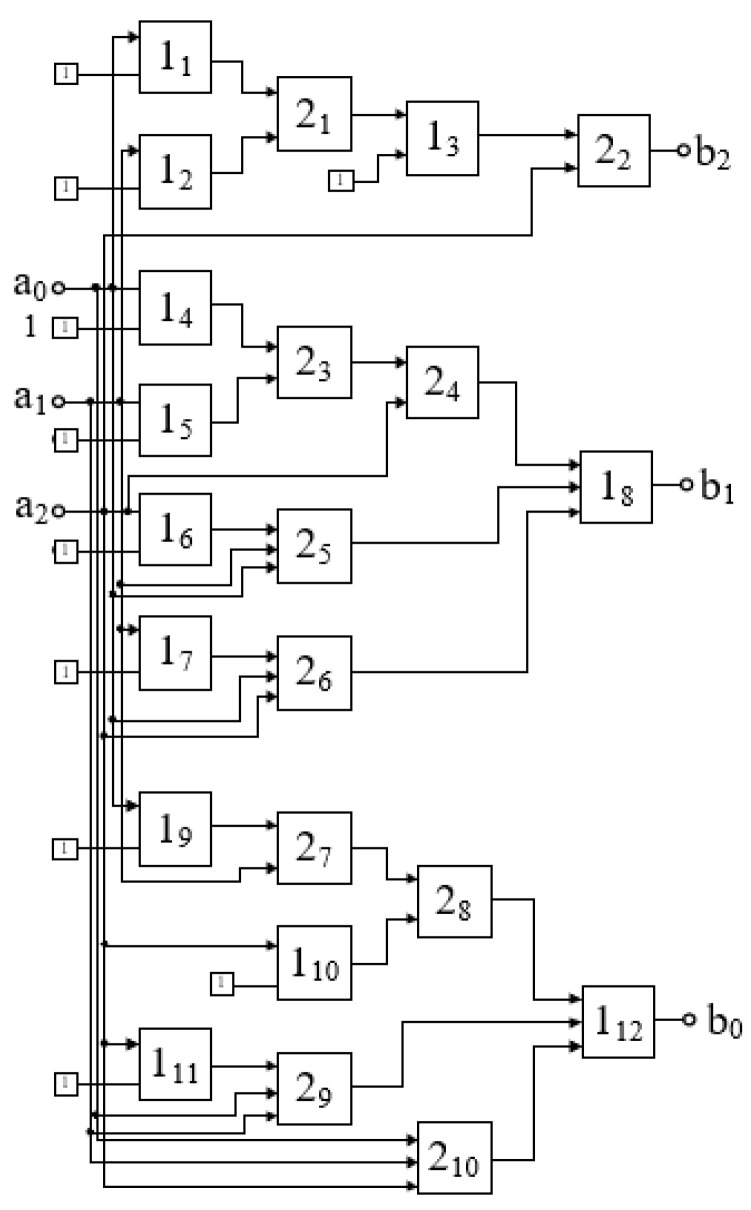
Schematic diagram of the digital logarithm unit: elements 1_n_—XOR; 2_n_—AND.

**Figure 6:**
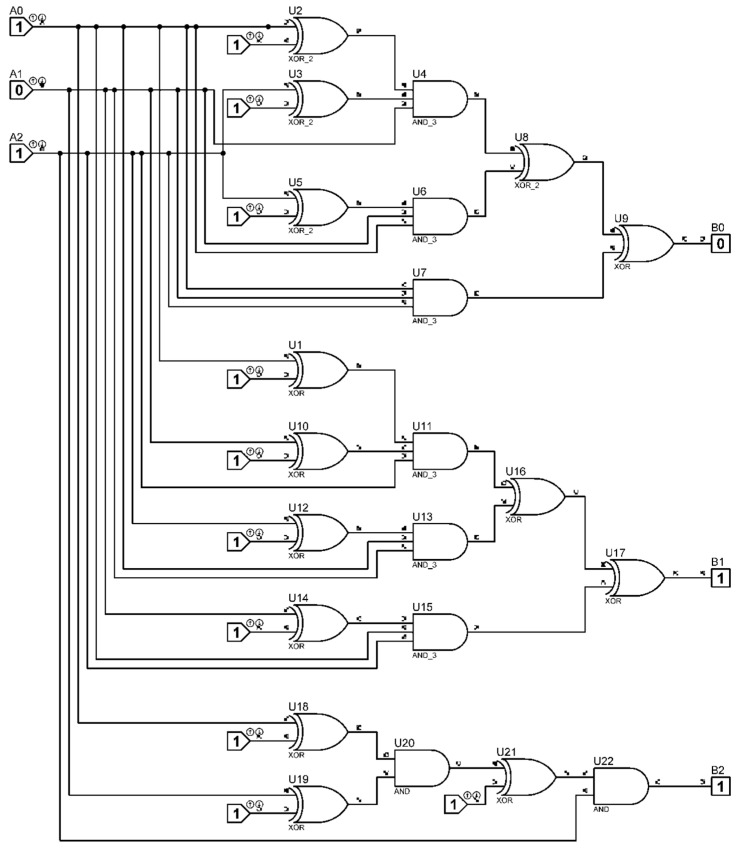
Implementation of the digital logarithm block by software Proteus.

The schematic diagram of the unit that implements these operations is shown in Figure 5. This scheme has been worked out in the Proteus 8.17 software environment (Figure 6). The test results confirmed the performance of this scheme. It should be noted that this block is key to the implementation of the proposed approach as a whole, since it is the operation of digital logarithm in accordance with Formula (14) that makes it possible to move from a rotating image to a still one. To accomplish this, it is enough to provide the calculation of the difference of the form

(40) $$\Delta LD=n_{2}LD\left[a_{n_{1}}(t)\right]-n_{1}LD\left[a_{n_{2}}(t)\right]$$

where $a_{n_{i}}(t)$ is the amplitude of the $n_{i}$-th spectral component at time t.

According to Formula (14), this value is expressed as

(41) $$∆LD=n_{2}LD\left[a_{n_{1}}^{0}\right]-n_{1}LD\left[a_{n_{2}}^{0}\right]$$

The expression in the right-hand side of Formula (41) is time-independent, which is proved by the above statement.

To verify the functionality of the hardware implementation of computational blocks 1–5 (Figure 3), a dedicated testbench was developed in VHDL using Quartus Prime and executed in the ModelSim simulation environment. Unlike manual selective testing, the virtual testbench performs an exhaustive enumeration of all possible input conditions. A total of 128 combinations of bits U_1_…U_7_, are sequentially applied to the sensor ports, and the computation results are sets of values b2b1b0, corresponding to the execution of the digital logarithm operation, which is a key element in implementing the proposed approach.

Within the testbench, a numerical model is implemented that fully corresponds to Formulas (27) through (39) presented above.

**Figure 7:**
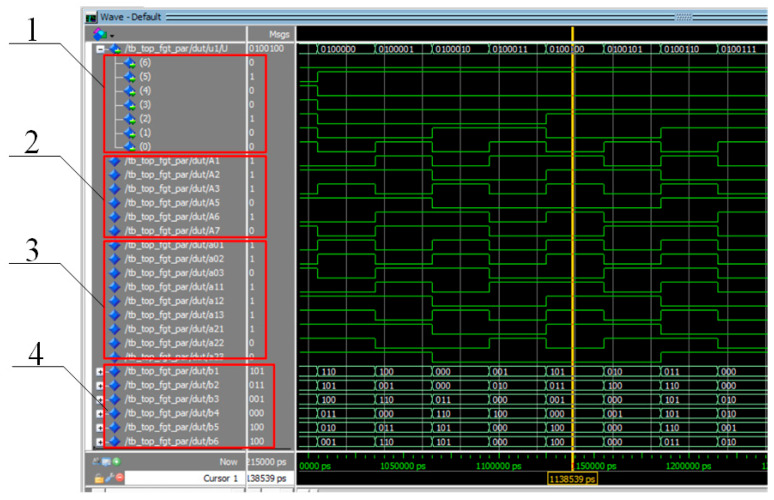
Computation results in the Waveform panel; ModelSim 10.5b environment: 1—input bits corresponding to the sensor states U_n_; 2—bits representing the binary values A_k_; 3—bits corresponding to the values $a_{im}$; 4—bits representing the output results of the digital logarithm block b_i_.

The timing diagram in Figure 7 illustrates the operational characteristics of the test bench. For each new set of values *U_i_*, the registered bits *A*_1_…*A*_7_ appear first, followed by the formation of the coefficients $a_{im}$. As a result, the entire set of output values *b*_2_*b*_1_*b*_0_, corresponding to the digital logarithm operation, is produced within a single clock cycle. A direct comparison of the results obtained using the presented test and those derived from tabular computations confirms the adequacy of the hardware implementation. The utilized software code is provided in Supplementary Materials.

This demonstrates that the described hardware algorithm produces results consistent with the original analytical expressions across the entire set of possible input states.

It should also be emphasized that the developed code can be directly utilized for the fabrication of any device based on the analysis of a rotating digital image of the specified type. In particular, field-programmable gate arrays (FPGAs) are now widely used. These chips are programmed using tools such as Quartus Prime 24.1std. The software code employed for testing can be converted into code suitable for FPGA operation using ModelSim. Thus, the developed software effectively replaces the need for designing most of the electronic components of devices of the proposed type.

## 4. Discussion

Thus, as demonstrated by the materials presented above, the analysis of digitally rotating images is most effectively performed using Fourier–Galois transforms along with the digital logarithm operation. This approach enables the reduction of a rotating image to a stationary one while simultaneously determining the rotation speed or frequency.

In cases where the digitized image has a ring-like structure and adheres to binary logic, it is advisable to employ Galois fields, which are algebraic extensions of the field GF(2). Although this method imposes certain constraints on the number of elements (pixels) into which the analysed image can be partitioned, it allows for a highly efficient construction of basis functions that facilitate the transformation of the Fourier–Galois spectrum of a rotating image into that of a stationary one.

Furthermore, the findings of this study indicate that, based on the basis functions derived from algebraic extensions of GF(2), it is possible to construct various basis systems that serve as analogues of the Walsh basis. Transitions between such systems of basis functions may be achieved through direct verification of orthogonality. While this method still requires further theoretical justification, the ongoing relevance of modernizing the Walsh basis warrants increased scholarly attention.

The expediency of further development of the proposed approach is also reflected in the example of a viscometer based on the analysis of circular images reduced to a discrete form. It should be emphasized that in addition to the traditional function performed by viscometers, the use of an image recognition system also makes it possible to study various types of mechanochemical reactions that can occur in the region close to the conditions when a phase transition occurs, accompanied by changes in the optical density of the solution.

The Galois field-oriented viscometer circuit is, of course, nothing more than a particular example of measuring instruments of this kind. We have used it mainly to demonstrate the possibilities of using algebraic extensions of the binary Galois field to recognize images that are themselves mapped in terms of binary logic (dark/light pixels). In addition, we show that electronic circuits that perform the operation of digital logarithm can be built on the basis of a fully formalized procedure based on the use of the Zhegalkin polynomial too.

A similar approach can be employed in the development of devices for various other applications based on digital image recognition. For instance, digital processing of droplet images on different substrates can be reliably used to investigate surface tension. The digital analysis of hydrogel sample images—including in dynamic mode—enables the extraction of information about swelling kinetics (in many studies, goniometers are still used for these purposes [51,52]).

The use of colour indicators (e.g., phenolphthalein) allows for the visualization of spatial heterogeneity in pH distribution, and there are also well-established methods for the experimental detection of non-uniform temperature distributions [53,54], among others. In addition, a wide variety of processes involve ions that actively interact with light in the optical range (“coloured” ions, such as anions formed by the dissociation of potassium hexacyanoferrate) [55,56].

As shown in [57], it is possible to propose an entire class of registration devices based on digital image processing, particularly for visualizing inhomogeneous flows arising in polymer hydrogel systems. Moreover, there are numerous opportunities to convert conventional measurement methodologies into procedures based on digital image analysis. For example, a large body of research has used turbidimetry to study phase transitions in solutions of thermoresponsive polymers [58,59,60]. To adapt such methodologies to digital image-based techniques, it is sufficient to employ cuvettes with an artificially induced temperature gradient.

It should also be emphasized that in the materials of this work, objects were considered that have only one well-defined type of symmetry (symmetry with respect to rotation around the selected axis). In the future, it can also be extended to objects with other types of symmetry (in particular, this applies to the digital processing of images of swelling hydrogels), and we do not necessarily have to talk about circular images, showing that in many cases it is advisable to switch to Galois coordinates, in which the elements of the Galois field (or its algebraic extension) correspond to the discrete coordinates of a pixel. As it was shown in [61], other algebraic structures, for example, algebraic rings, can be used for a similar purpose. In the cited works, the construction of Galois coordinates similar to Cartesian ones was considered. However, it is also possible to construct Galois coordinates similar to polar ones in the future. There are additional opportunities for analysing images of various types, since if the flat image is finite, then an analogue of any of the above coordinate systems can be used (depending on the specifics of the problem to be solved).

## 5. Conclusions

Thus, the digital logarithm operation serves as a highly convenient tool for analysing rotating ring-shaped images segmented into pixels whose states are described by binary logic variables. This operation enables the transition from the analysis of rotating images to that of stationary ones by leveraging the spectral characteristics of digital functions obtained via the Fourier–Galois transform.

An example of a task requiring the analysis of such images is presented by a viscometer that registers the rotational motion of a bead in a viscous medium under investigation.

For images of this type, it is appropriate to employ the Fourier–Galois transform, whose basis functions are constructed through classical algebraic extensions of the Galois field GF(2), corresponding to binary logic. This approach also facilitates the implementation of electronic circuits that directly perform the digital logarithm operation.

For each specific image type containing $n=2^{p}-1\;$pixels, the formulas necessary to execute the digital logarithm operation can be explicitly derived using Zhegalkin polynomials. In the present work, this approach is utilized to design the electronic circuitry of the aforementioned viscometer.

## Figures and Tables

**Table 1:** The elements of the field GF(2^3^) are the powers of the primitive element θ.

*n*	0	1	2	3	4	5	6
$\theta^{n}$	1	θ	$\theta^{2}$	θ+1	$\theta^{2}+\theta$	$\theta^{2}+\theta +1$	$\theta^{2}+1$

**Table 2:** Degrees of the primitive element corresponding to the elements of the sequences $g_{n}(m)$.

	*m* = 1	2	3	4	5	6	0
*n* = 1	1	2	3	4	5	6	0
2	2	4	6	1	3	5	0
3	3	6	2	5	1	4	0
4	4	1	5	2	6	3	0
5	5	3	1	6	4	2	0
6	6	5	4	3	2	1	0
7	0	0	0	0	0	0	0

**Table 3:** Basis sequences $g_{n}(m)$ in binary representation corresponding to Formula (16).

n=1								
$u_{1m}$	θ	$\theta^{2}$	θ+1	$\theta^{2}+\theta$	$\theta^{2}+\theta +1$	$\theta^{2}+1$	1	s
m	1	2	3	4	5	6	0	
$z_{0}$	0	0	1	0	1	1	1	3
$z_{1}$	1	0	1	1	1	0	0	1
$z_{2}$	0	1	0	1	1	1	0	2
n=2								
$u_{1m}$	$\theta^{2}$	$\theta^{2}+\theta$	$\theta^{2}+1$	θ	θ+1	$\theta^{2}+\theta +1$	1	s
m	2	4	6	1	3	5	0	
$z_{0}$	0	0	1	0	1	1	1	3
$z_{1}$	0	1	0	1	1	1	0	2
$z_{2}$	1	1	1	0	0	1	0	4
n=3								
n=3	n=3	n=3	n=3	n=3	n=3	n=3	n=3	n=3
n=3	n=3	n=3	n=3	n=3	n=3	n=3	n=3	
n=3	n=3	n=3	n=3	n=3	n=3	n=3	n=3	n=3
n=3	n=3	n=3	n=3	n=3	n=3	n=3	n=3	n=3
n=3	n=3	n=3	n=3	n=3	n=3	n=3	n=3	n=3
n=4								
$u_{1m}$	$\theta^{2}+\theta$	θ	$\theta^{2}+\theta +1$	$\theta^{2}$	$\theta^{2}+1$	θ+1	1	s
m	4	1	5	2	6	3	0	
$z_{0}$	0	0	1	0	1	1	1	3
$z_{1}$	1	1	1	0	0	1	0	4
$z_{2}$	1	0	1	1	1	0	0	1
n=5								
$u_{1m}$	$\theta^{2}+\theta +1$	θ+1	θ	$\theta^{2}+1$	$\theta^{2}+1$	$\theta^{2}$	1	s
m	5	3	1	6	4	2	0	
$z_{0}$	1	1	0	1	0	0	1	7
$z_{1}$	1	1	1	0	1	0	0	6
$z_{2}$	1	0	0	1	1	1	0	8
n=6								
$u_{1m}$	$\theta^{2}+1$	$\theta^{2}+\theta +1$	$\theta^{2}+\theta$	θ+1	$\theta^{2}$	θ	1	s
m	6	5	4	3	2	1	0	
$z_{0}$	1	1	0	1	0	0	1	7
$z_{1}$	0	1	1	1	0	1	0	5
$z_{2}$	1	1	1	0	1	0	0	6

**Table 4:** Orthogonality properties of the obtained functions $z_{i}^{n}(m)$.

*s*	1	2	3	4	5	6	7	8
1	0	0	0	0	0	1	0	1
2	0	0	0	0	1	0	0	1
3	0	0	0	0	0	0	1	0
4	0	0	0	0	1	1	0	0
5	0	1	0	1	0	0	0	0
6	1	0	0	1	0	0	0	0
7	0	0	1	0	0	0	0	0
8	1	1	0	0	0	0	0	0

**Table 5:** Values of coefficients $\alpha_{km}$ in Formula (26).

	*m* = 1	2	3	4	5	6
*k* = 1	θ	$\theta^{2}$	0	$\theta +\theta^{2}$	0	0
2	$\theta^{2}$	$\theta +\theta^{2}$	0	θ	0	0
3	1	1	0	1	0	0
5	0	0	$\theta +\theta^{2}$	0	$\theta^{2}$	θ
6	0	0	θ	0	$\theta +\theta^{2}$	$\theta^{2}$
7	0	0	1	0	1	1

**Table 6:** Values of coefficients $\beta_{ikm}$ in Formula (34).

i=0						
	*m* = 1	2	3	4	5	6
*k* = 1	0	0	0	0	0	0
2	0	0	0	0	0	0
3	1	1	0	1	0	0
5	0	0	0	0	0	0
6	0	0	0	0	0	0
7	0	0	1	0	1	1
i=1						
	*m* = 1	2	3	4	5	6
*k* = 1	1	0	0	1	0	0
2	0	1	0	1	0	0
3	0	0	0	0	0	0
5	0	0	1	0	0	1
6	0	0	1	0	1	0
7	0	0	0	0	0	0
i=2						
	*m* = 1	2	3	4	5	6
*k* = 1	0	1	0	1	0	0
2	1	1	0	0	0	0
3	0	0	0	0	0	0
5	0	0	1	0	1	0
6	0	0	0	0	1	1
7	0	0	0	0	0	0

**Table 7:** The values of the binary variables used for the digital logarithm operation.

*k*	1	2	3	4	5	6	0
*a*_0_	0	0	1	0	1	1	1
*a*_1_	1	0	1	1	1	0	0
*a*_2_	0	1	0	1	1	1	0
*b*_0_	1	0	1	0	1	0	0
*b*_1_	0	1	1	0	0	1	0
*b*_2_	0	0	0	1	1	1	0

## Data Availability

The original contributions presented in this study are included in the article. Further inquiries can be directed to the corresponding authors.

## Supplementary Materials

The following supporting information can be downloaded at: https://www.mdpi.com/article/10.3390/polym17131791/s1, TestBench_code.docx, Patent.
