# Supplementary material for: Application of Fourier-Galois Spectra Analysers for Rotating Image Analysis
Source: Polymers (Basel). 2025 Jun 27;17(13):1791. doi: 10.3390/polym17131791 (PMC12252000; doi:10.3390/polym17131791)
Supplement: Supplementary file 1 [file polymers-17-01791-s001.zip › TestBench_code.pdf]

```
library ieee;
use ieee.std_logic_1164.all;
use ieee.numeric_std.all;

entity tb_top_fgt_par is end;

architecture sim of tb_top_fgt_par is
   constant Tclk : time := 10 ns;
   signal clk    : std_logic := '0';
   signal U_raw  : std_logic_vector(6 downto 0);
   signal bout   : std_logic_vector(17 downto 0);
   signal fresh  : std_logic;


   function gf_log(a : std_logic_vector(2 downto 0))
         return std_logic_vector is
      type vec3 is array(0 to 7) of std_logic_vector(2 downto 0);
      constant lut : vec3 :=
         ( "000","000","001","011","010","110","100","101" );
   begin
      return lut(to_integer(unsigned(a)));
   end;


   function ref_log(U : std_logic_vector(6 downto 0);
               col : integer)
         return std_logic_vector is
      variable u1,u2,u3,u4,u5,u6,u7 : std_logic;
      variable A1,A2,A3,A5,A6,A7   : std_logic;
      variable c2,c1,c0            : std_logic;
   begin
      u1:=U(0); u2:=U(1); u3:=U(2); u4:=U(3); u5:=U(4); u6:=U(5); u7:=U(6);

      A1:=u1 xor u3 xor u4 xor u5;
      A2:=u2 xor u4 xor u5 xor u6;
      A3:=u1 xor u5 xor u6 xor u7;
      A5:=u2 xor u3 xor u4 xor u6;
      A6:=u1 xor u2 xor u3 xor u5;
      A7:=u1 xor u2 xor u4 xor u7;

      case col is
         when 0 => c2:=A2;           c1:=A1;           c0:=A3;
         when 1 => c2:=A1 xor A2;    c1:=A2;           c0:=A3;
         when 2 => c2:=A5;           c1:=A5 xor A6;    c0:=A7;
         when 3 => c2:=A1 xor A6;    c1:=A1 xor A2;    c0:=A3;
         when 4 => c2:=A5 xor A6;    c1:=A6;           c0:=A7;
         when 5 => c2:=A6;           c1:=A5;           c0:=A7;
         when others => c2:='X';     c1:='X';          c0:='X';
      end case;
      return gf_log(c2 & c1 & c0);
   end;
```

```
   function ref6(U : std_logic_vector(6 downto 0))
           return std_logic_vector is
      variable v : std_logic_vector(17 downto 0);
   begin

      v(17 downto 15) := ref_log(U,5);
      v(14 downto 12) := ref_log(U,4);
      v(11 downto  9) := ref_log(U,3);
      v( 8 downto  6) := ref_log(U,2);
      v( 5 downto  3) := ref_log(U,1);
      v( 2 downto  0) := ref_log(U,0);
      return v;
   end;

begin
   ------------------------------------------------------------------
   clk <= not clk after Tclk/2;

   dut : entity work.top_fgt_par
      port map (clk=>clk, U_raw=>U_raw, b_out=>bout, fresh=>fresh);

   ------------------------------------------------------------------

   stim : process
   begin
      wait for 3*Tclk;
      for vec in 0 to 127 loop
         U_raw <= std_logic_vector(to_unsigned(vec,7));
         wait for 3*Tclk;
      end loop;
      wait;
   end process;

   ------------------------------------------------------------------

   check : process
   begin
      wait until rising_edge(fresh);
      assert bout = ref6(U_raw)
        report "Mismatch U=" & integer'image(to_integer(unsigned(U_raw)))
        severity error;
   end process;
end;
```
