# Supplementary material for: Application of Fourier-Galois Spectra Analysers for Rotating Image Analysis
Source: Polymers (Basel). 2025 Jun 27;17(13):1791. doi: 10.3390/polym17131791 (PMC12252000; doi:10.3390/polym17131791)
Supplement: Supplementary file 1 [file polymers-17-01791-s001.zip › Viscometer.pdf]

ҚАЗАҚСТАН РЕСПУБЛИКАСЫ РЕСПУБЛИКА КАЗАХСТАН

REPUBLIC OF KAZAKHSTAN

ПАТЕНТ
PATENT

№ 4278

ПАЙДАЛЫ МОДЕЛЬГЕ / НА ПОЛЕЗНУЮ МОДЕЛЬ / FOR UTILITY MODEL

(21) 2019/0582.2

(22) 21.06.2019

Қазақстан Республикасы Пайдалы модельдер мемлекеттік тізілімінде тіркеу күні / Дата регистрации в Государственном реестре полезных моделей Республики Казахстан / Date of the registration in the State Register of Utility Models of the Republic of Kazakhstan: 04.09.2019

(54) Вискозиметр
Вискозиметр
Viscometer

(73) Мун Григорий Алексеевич (KZ); Сулейменов Ибрагим Эсенович (KZ); Байпакбаева Салтанат Туркестанкызы (KZ)
Mun Grigoriy Alekseyevich (KZ); Suleimenov Ibragim Esenovich (KZ); Baipakbayeva Saltanat Turkestankyzy (KZ)

(72) Сулейменов Ибрагим Эсенович (KZ)
Мун Григорий Алексеевич (KZ)
Копишев Эльдар Ертаевич (KZ)
Байпакбаева Салтанат Туркестанкызы (KZ)
Шалтыкова Дина Бернаровна (KZ)
Кабдушев Шернияз Булатулы (KZ)

Suleimenov Ibragim Esenovich (KZ)
Mun Grigoriy Alekseyevich (KZ)
Kopishev Eldar Yertayevich (KZ)
Baipakbayeva Saltanat Turkestankyzy (KZ)
Shaltykova Dina Bernarovna (KZ)
Kabdushev Sherniyaz Bulatuly (KZ)

ЭЦҚ қол қойылды
Подписано ЭЦП
Signed by EDS

Е. Оспанов
Y. Ospanov

«Ұлттық зияткерлік меншік институты» РМК директоры
Директор РГП «Национальный институт интеллектуальной собственности»
Director of the «National Institute of Intellectual Property» RSE

ҚР ӘМ «Ұлттық зияткерлік меншік институты» РМК
РГП «Национальный институт
интеллектуальной собственности» МЮ РК
National Institute of Intellectual Property,
Ministry of Justice of the Republic of Kazakhstan

Нұр-Сұлтан қаласы, Қорғалжын тас жолы, 3Б ғимараты
город Нур-Султан, шоссе Коргалжын, здание 3Б
Nur-Sultan, Korgalzhyn highway, 3B Building
Телефон / Telephone number: +7 (7172) 62-15-15

E-mail: kazpatent@kazpatent.kz
http:// www.kazpatent.kz

Патентті күшінде ұстау ақысы уақытылы төленген жағдайда,
патенттің күші Қазақстан Республикасының бүкіл аумағында қолданылады.
Действие патента распространяется на всю территорию Республики Казахстан
при условии своевременной оплаты поддержания патента в силе.
Subject to timely payment for the maintenance of the patent in force
the effect of the patent extends to the entire territory of the Republic of Kazakhstan.

«ҰЗМИ» РМК веб - порталында Қазақстан Республикасы Пайдалы модельдер мемлекеттік
тізілімі белімінде пайдалы модель патентіне толық сипаттамасы қолжетімді.
Полное описание полезной модели к патенту
доступно на веб-портале РГП «НИИС» в разделе «Государственные реестры
полезных моделей Республики Казахстан».
Full description of the patent is available on the NIIP web portal in the State Register of Utility Models
of the Republic of Kazakhstan section.
